# Single-cell analyses reveal the therapeutic effects of ATHENA and its mechanism in a rhabdomyosarcoma patient

**DOI:** 10.3389/fonc.2022.1039145

**Published:** 2022-11-29

**Authors:** Yujun Liu, Ke Wang, Yanli Zhou, Xibing Zhuang, Shali Shao, Fulu Qiao, Xiangdong Wang, Xin Zou, Tiankui Qiao

**Affiliations:** ^1^ Center for Tumor Diagnosis and Therapy, Jinshan Hospital, Fudan University, Shanghai, China; ^2^ Nuclear Medicine Department, Jinshan Hospital, Fudan University, Shanghai, China; ^3^ Department of Breast Surgery, Tai’an Central Hospital, Tai’an, China

**Keywords:** single-cell RNA sequencing, autologous tumor vaccines, scSTAR, T cells, B cells, exhausted state, central memory

## Abstract

**Background:**

Whole-cell tumor vaccines tend to suffer from low immunogenicity. Our previous study showed that irradiated lung cancer cell vaccines in mouse models enhance antitumor efficacy by eliciting an intensive T cells response and improving immunogenicity. Based on these findings, we developed an improved whole-cell tumor vaccine, Autologous Tumor Holo antigEn immuNe Activation (ATHENA).

**Methods:**

In this study, we report the successful treatment of a 6-year-old male diagnosed with meningeal rhabdomyosarcoma with pulmonary and liver metastases using ATHENA. After 6 cycles of therapy, PET/CT showed the therapeutic efficacy of ATHENA. We profiled the immune response by single-cell RNA sequencing (scRNA-seq). Flow cytometry analysis was implemented to validate the status transitions of CD8^+^ T cells.

**Results:**

In CD8^+^ T cells, the exhausted status was weakened after treatment. The exhausted CD4^+^ T cells shifted towards the central memory phenotype after the treatment. Breg cells were converted to Plasma or Follicular B cells. Survival analysis for pan-cancer and transcription factor analysis indicated that such T cell and B cell transitions represent the recovery of antitumoral adaptive immune response. We validated that the proportion of CD279^+^CD8^+^ T cells were reduced and the expression of CD44 molecule was upregulated by flow cytometry assay.

**Conclusion:**

Such studies not only show that ATHENA therapy may be a promising alternative treatment for tumor patients but provide a novel idea to analyses the mechanisms of rare cases or personalized cancer treatment.

## Introduction

Since the introduction of immune checkpoint blockade and chimeric antigen receptor (CAR)-T cells, cancer immunotherapies have played a vital role in treating cancer (1, 2). Cancer vaccines have been a promising immunotherapeutic approach with preventive and therapeutic potential, aiming to induce tumor regression, eradicate microscopic residual disease, establish a lasting anti-tumor memory and avoid adverse effects (3). There are four categories of tumor vaccines under investigation, including peptide- and protein-based vaccines, cellular vaccines, genetic vaccines and other types of cancer vaccines (4). Autologous whole-cell cancer vaccines are a type of cellular vaccines prepared to utilize the patient’s own tumor cells. Compared to other immunotherapies targeting specific antigens, it has the advantage of carrying relatively intact tumor antigens and this allows the activation of CD4^+^ T helper and CD8^+^ cytotoxic lymphocytes simultaneously (5–7). However, vaccines made from inactivated tumor cells are extremely weak in immunogenicity and do not induce sufficient antitumor immune effects (8, 9). Therefore, it is crucial to find novel strategies for enhancing vaccine immunogenicity.

Radiotherapy has been closely related to immunotherapy. In contrast with conventional fractionated radiotherapy, stereotactic body radiotherapy (SBRT) is a high-dose, low-frequency technique that delivers high doses of radiation therapy precisely to small volume targets in a single or small number of treatments (10, 11). Recent studies have shown that SBRT can mediate immunostimulatory effects by upregulating the expression levels of immunogenic cell surface markers and promoting the exposure and release of tumor-associated antigens (TAAs) (12, 13). In previous studies, ionizing radiation (IR) was only used to inactivate tumor cells to prepare vaccines, usually with a lethal radiation dose of 20-200 Gy (14–16). Our previous studies showed that irradiated Lewis lung cancer (LLC) vaccines had a significantly stronger antitumor effect in mice bearing LLC xenografts than LLC vaccines. We observed that a high dose of irradiation not only triggers the release of many TAAs and cytokines but also that their radiated tumor cells show higher antigenicity (11, 17). We named the therapy ATHENA. Here, we report a successful case of a 6-year-old male diagnosed with meningeal rhabdomyosarcoma with pulmonary and liver metastases. ATHENA treatment showed dramatic antitumor effects. According to the results of PET/CT, the liver metastases and the nodal reduction of lung metastases completely disappeared. The patient was evaluated as having a complete response (CR) by RECIST criteria (18).

Herein, we applied scRNA-seq to comprehensively profile the dynamic changes in peripheral immune circulation from patients before and after ATHENA treatment. We discovered that ATHENA might restore the adaptive immune response to produce antitumor effects by reversing the exhausted phenotype toward the effector memory phenotype and stimulating activation of B cells.

## Materials and methods

### Vaccines preparation

The fresh tumor tissue was obtained after surgical resection from a patient diagnosed with meningeal rhabdomyosarcoma with pulmonary and liver metastases. Then, the tumor tissue was irradiated using a Trilogy linear accelerator (Varian Medical Systems, CA, USA) and cultured in DMEM (Sigma, Louis, Missouri, USA) with 10% FBS (# 35–015-CV) for 24h. For preparation of tumor cell lysates (TCLs), the irradiated tissue was digested in trypsin and suspended in 0.9% saline, subjected to five cycles of freezing, thawing (liquid nitrogen for 5 minutes, 37°C for 5 minutes) and then centrifuged at 1,500 rpm for 10 minutes. Afterwards, the supernatants were collected through a 0.22mum membrane filter and stored at -80°C.

### Human Specimen collection and processing

Human peripheral blood was obtained before and after treatment from a patient diagnosed with meningeal rhabdomyosarcoma with pulmonary and liver metastases. The patient was treated with 6 cycles of subcutaneous injections of interleukin-2 (50×10^4^ IU) on the first and second days and 1 mL of irradiated autologous tumor whole-cell component vaccines on the second day. Clinical response was assessed for each target lesion based on RECIST v.1.1 (18). This study was approved by the Ethics Committee of the Jinshan Hospital of Fudan University (Approved ID: JIEC-2019-09). Informed consent was signed by the enrolled patient. Fresh peripheral blood was collected at baseline and 1 week after treatment initiation in EDTA anticoagulant tubes and subsequently layered onto Lymphocyte Separation Medium (LTS1077, TBD). After centrifugation, lymphocyte cells remained at the plasma–Lymphocyte-H interface and were carefully transferred to a new tube and washed twice with 1x phosphate-buffered saline (PBS, Sangon). After the supernatant was removed, the pelleted cells were suspended in red blood cell lysis buffer (LEAGENE) and left to lyse for 5 min at room temperature, followed by further 1x PBS washes. Then, the cells were cryopreserved for subsequent experiments. Before experiments were initiated, cells were thawed and counted. Viable cell yield was determined by Trypan Blue exclusion. After passing the test, library prep was performed using the manufacturer’s instructions (10X Genomics).

### scRNA-seq data generation

Beads with unique molecular identifiers (UMIs) and cell barcodes were loaded close to saturation so that each cell was paired with a bead in a gel beads-in-emulsion (GEM). After exposure to cell lysis buffer, polyadenylated RNA molecules hybridized to the beads. Beads were retrieved into a single tube for reverse transcription. On cDNA synthesis, each cDNA molecule was tagged on the 5’ end with UMI and cell label indicating its cell of origin. Briefly, 10X Genomics beads were subjected to second-strand cDNA synthesis, adaptor ligation, and universal amplification. Sequencing libraries were prepared using randomly interrupted whole-transcriptome amplification products to enrich the 3’ end of the transcripts linked with the cell barcode and UMI. All the remaining procedures, including library construction, were performed according to the standard manufacturer’s protocol (CG000206 RevD). Sequencing libraries were quantified using a High Sensitivity DNA Chip (Agilent) on a Bioanalyzer 2100 and the Qubit High Sensitivity DNA Assay (Thermo Fisher Scientific). The libraries were sequenced on a NovaSeq6000 (Illumina) using 2x150 chemistry.

### scRNA-seq data processing, quality control and integration

The FASTQ files were generated by using the 10X Genomics Cell Ranger toolkit (v.3.0.1), which extracted barcodes and UMI, filtered and mapped reads to the GRCh38 reference genome, and obtained a matrix containing normalized gene counts versus cells per sample. Then, we further analyzed these outputs using Seurat (v.4.1.2) for quality control and downstream analysis with default parameters, unless otherwise indicated. We removed out low-quality cells according to the following quality control procedures (1): cells with fewer than 200 genes or more than 3000 genes and genes expressed in fewer than 3 cells were removed (2). Over 10% mitochondrial-derived UMI counts were filtered out. After quality control, the datasets were log-normalized. Since two samples from one patient before and after treatment were processed, we need to remove potential batch effects using the Seurat function “FindIntegrationAnchors” for dataset integration. The top 2000 highly variable genes (HVGs) were chosen for CCA, and the first 30 reduced dimensions were adopted for integration anchors. Finally, a total of 7467 single cells were used in downstream analyses.

### Dimensionality reduction, clustering, annotation and visualization

Then, the data were scaled using the ScaleData function. Principal component analysis (PCA) was performed, and we selected the top 30 PCs for clustering cells with a resolution parameter of 1. The same PCs were used to generate the uniform manifold approximation and projection (UMAP). Each cluster was annotated based on high expression of known marker genes and visualized by the FeaturePlot function, including CD3E, CD3D (T cells), CD8A, CD8B (CD8^+^ T cells), CD4, FOXP3 (CD4^+^ T cells and Treg cells), FCGR3A, NCAM1, CD3E (NK cells and NKT cells), CD19, CD79A, MS4A1 (B cells), CD163, CD68, CD14 (Myeloid cells), TRDC, CD3D (γδT cells), and PPBP (Platelet cells). The FindAllMarkers Function was used to validate the identification of differentially expressed marker genes for each cluster. To further identify immune cell subtypes at a high resolution, CD8^+^ T and CD4^+^ T cells were refined and reprojected separately with the same above methods applied to annotate the cell subtype. The two main clusters were subdivided into CD8^+^ T_CM_, CD8^+^ T_EX_, CD4^+^ T_EM_, CD4^+^ T_CM_, CD4^+^ T_EX1_ and CD4^+^ T_EX2_ based on states with clearly distinct differential expression profiles.

### scSTAR analysis

First, the normalized data were separated and remerged according to treatment time points. We used the OGFSC package to filter genes on the normalized data. Then, findAnchors and scSTAR functions were performed to obtain new gene expression data. Finally, we utilized the Seurat package (v.4.1.2) for downstream analysis.

### Differentially expressed genes and GSEA Pathway analysis

The FindAllMarker function in the Seurat package was used to obtain the differentially expressed genes in clusters using the Wilcoxon rank-sum test. A Bonferroni false discovery rate (FDR) correction less than 0.05 was used as a cutoff for identifying statistically significant DEGs. Then, we used GSEA (19, 20) to perform GO biological process enrichment analysis on the differentially expressed genes in each subset that was associated with before or after treatment. Gene sets with a significance level of FDR of < 0.05 were considered significant.

### Flow cytometry analysis

Up to 1x10^6^ dissociated WBCs were resuspended in Zombie Aqua Fixable Viability Kit (423101, Biolegend) (1:200 in PBS), followed by 15 minutes incubation at RT in the dark. Cells were then added to the same volume of FACS, centrifuged, resuspended in PBS and stained at 0.5-1x10^6^ cells/mL concentration with the following antibodies for 15 minutes at RT: Brilliant Violet 605TM anti-human CD3 (317321, Biolegend), Alexa Fluor 700 anti-human CD8 (344723, Biolegend), PE/Cyanine7 anti-human CD279 (PD-1) (621615, Biolegend) and FITC anti-human CD44 (338803, Biolegend). Stained cells were analyzed with flow cytometer (BD Biosciences).

### Trajectory analysis

To analyze the trajectory of CD8^+^ T, CD4^+^ T, B and Myeloid cells based on scRNA-seq expression data, we utilized Moncle2 (21) to determine the potential lineage differentiation. All functions were performed with default parameters to characterize the data.

### Survival analysis

A standard Kaplan-Meier survival analysis (22) for 21 cancer types was used to analyze the association of these clusters processed by scSTAR with overall survival (OS).

### SCENIC analysis

SCENIC (23) analysis was developed to assess the regulatory network utilizing the motifs database for RcisTarget and Genie3. The seed was set to 777. The normalized expression data were input into SCENIC (version 1.3.1) to build co-expression modules. Then, a motif dataset (hg38:refseq-r80:500bp_up_and_100bp_down_tss.mc9nr. feather and hg38:refseq-r80:10kb_up_and_down_tss.mc9nr.feather) was used to construct regulons for each TF in SCENIC. Finally, the regulon activity score (RAS) was calculated by the AUCell package.

### Statistical analysis

All statistical analyses were performed using R (version 4.1.2). A two-sided Wilcoxon rank-sum test with Bonferroni FDR correction was used for all differential expression analyses between clusters.

## Results

### Analysis of single immune cell profiling after ATHENA treatment

**Figure 1:**
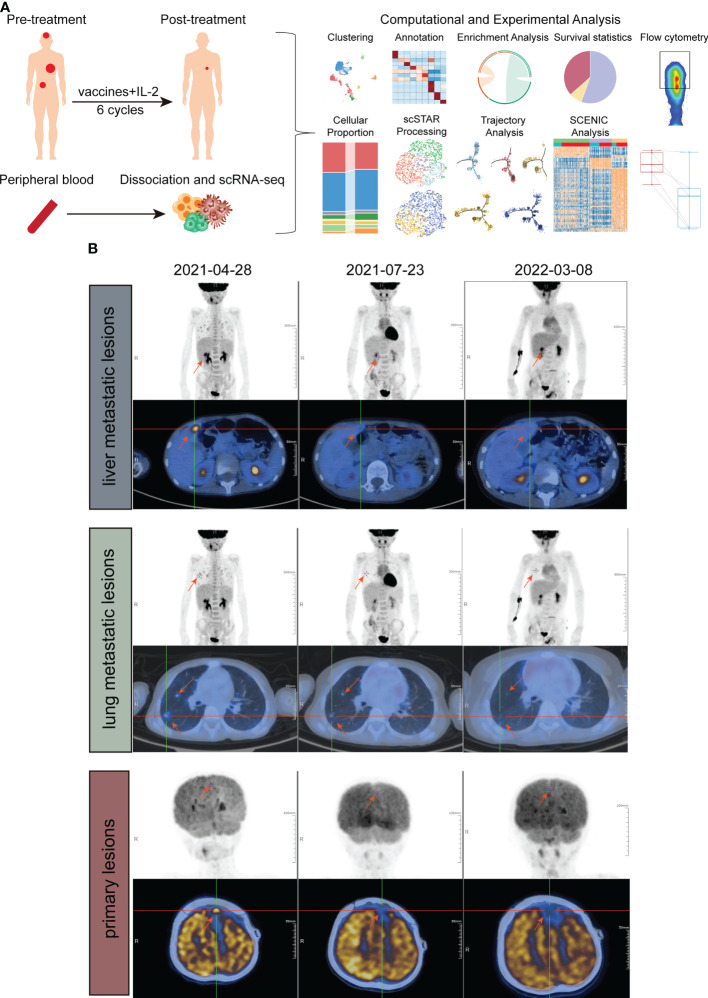
Study design and examinations of patients in different periods. **(A)** Schematic of the experimental design and analytical work. **(B)** PET/CT imaging of liver metastatic lesions (above), lung metastatic lesions (middle) and primary lesion (down) at three timepoints, before treatment, approximately 6 weeks after treatment and approximately 1 year after treatment.

**Figure 2:**
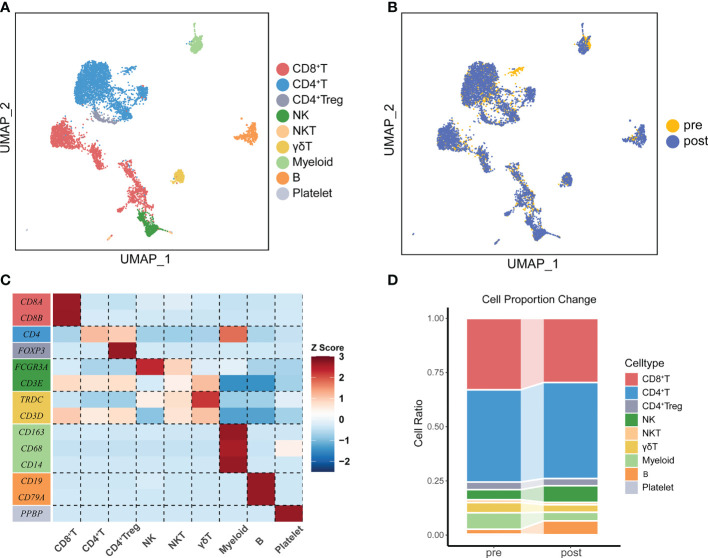
The immune landscape of PBMCs from patients treated with ATHENA. **(A)** Uniform manifold approximation and projection (UMAP) of all PBMCs before and after therapy. Cells are colored based on 9 clusters defined by k-means clustering, which include the CD8^+^ T cell cluster, CD4^+^ T cell cluster, CD4^+^ Treg cell cluster, NK cell cluster, NKT cell cluster, γδ T cell cluster, Myeloid cell cluster, B cell cluster and Platelet cell cluster. **(B)** UMAP of all PBMCs, colored according to ATHENA treatment history. **(C)** The expression patterns of canonical markers in distinct immune cell clusters. **(D)** Sankey diagram showing the percentage of cells per sample for clusters before and after treatment.

One patient diagnosed with left frontal rhabdomyosiform with multiple metastases in both lungs and metastases in the S4 segment of the liver received 6 cycles of ATHENA treatment. Blood samples were collected at pre-treatment and post-treatment immediately (**Figures 1A, B**). We performed droplet-based 5’ scRNA-seq sequencing to profile the PBMC samples. After quality control, we obtained single-cell transcriptome data for 7449 high-quality immune cells. Then, performing unsupervised clustering and uniform manifold approximation and projection (UMAP) plot analyses, we utilized gene expression patterns of canonical markers to classify the 20 clusters into 9 major cell populations that included CD8^+^ T cells (2281, 30.55%), CD4^+^ T cells (3271, 43.80%), CD4^+^ Treg cells (253, 3.39%), NK cells (508, 6.80%), NKT cells (86, 1.15%), γδT cells (286, 3.83%), myeloid cells (374, 5.01%), B cells (390, 5.22%) and platelet cells (18, 0.24%) (**Figures 2A–C**). Compared with the pre-treatment sample, the post-treatment sample harbored a relatively higher proportion of CD4^+^ T cells (fold change of 1.04), NK cells (fold change of 1.65), and B cells (fold change of 2.79), while the proportions of CD8^+^ T cells (fold change of 0.90), CD4^+^ Treg cells (fold change of 0.95), NKT cells (fold change of 0.83), γδT cells (fold change of 0.73) and myeloid cells (fold change of 0.52) were lower than those in the pre-treatment samples (**Figure 2D**).

### Identification of T lymphocytes subsets by single-cell RNA-seq

**Figure 3:**
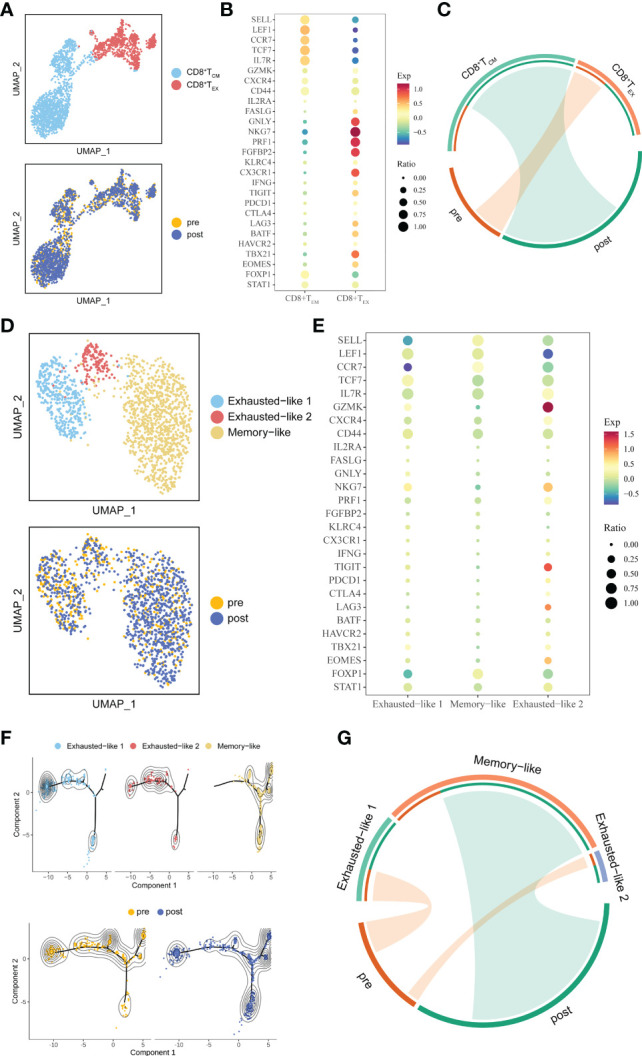
Detailed characterization of CD8^+^ T cells and their association with treatment. **(A)** UMAP of all CD8^+^ T cells, colored by cell type and sample. **(B)** Bubble plot of selected CD8^+^ T cell function-associated genes in each cell cluster. **(C)** The association between subtypes and treatment in CD8^+^ T cells. The area of the ties represents the relative enrichment. **(D)** UMAP of all CD8^+^ T_CM_ cells, colored by cell type and sample. **(E)** Bubble plot of selected CD8^+^ T_CM_ cells function-associated genes in each cell cluster. **(F)** Pseudotime analysis of CD8^+^ T_CM_ cells derived from PBMC samples inferred by Monocle2. Each point corresponds to an individual cell colored by cluster (above) or by treatment (down). The density curves represent the distribution of each cluster. **(G)** The association between subtypes and treatment in CD8^+^ T_CM_. The area of the ties represents the relative enrichment.

Because whole tumor cells are a good source of TAAs and can induce simultaneous CD8^+^ cytotoxic T lymphocytes (CTLs) and CD4^+^ T helper cell activation (7), we further unsupervised clustering CD8^+^ T cells and defined two sub-clusters: CD8^+^ T_CM,_ which highly expressed markers of memory-like phenotype (SELL, LEF1, CCR7, IL7R, TCF7, CD44, CXCR4), and CD8^+^ T_EX,_ which highly expressed the known exhausted markers (TIGIT, PDCD1, CTLA4, LAG3, HAVCR2, BATF) (24) (**Figures 3A, B**). The association between sub-clusters and pre- or post-treatment samples showed that CD8^+^ T_CM_ cells were enriched in post-treatment, while CD8^+^ T_EX_ cells had a strong association with pre-treatment (**Figure 3C**). Since trajectory analysis (21) did not clearly reflect changes in the two cell populations before and after treatment (**Figure S2A**), we further re-clustered CD8^+^ T_EX_ and CD8^+^ T_CM_. Three sub-clusters were identified in CD8^+^ T_CM,_ which named Exhausted-like 1, Exhausted-like 2 and Memory-like cells (**Figures 3D, E**). Trajectory analysis identified a main trajectory backbone, reflecting a possible state change (Exhausted-like 1 and Exhausted-like 2 to Memory-like cells) (**Figure 3F**). As we expected, Memory-like cells were enriched in post-treatment, while Exhausted-like 1 and Exhausted-like 2 cells had a strong association with pre-treatment (**Figure 3G**). Compared with CD8^+^ T_CM_, CD8^+^ T_EX_ was divided into 4 sub-clusters (PEX-like 1, PEX-like 2, TEX-like and Effector-like) and no obvious change in trajectory before and after treatment was observed (**Figures S2A–C**). Thus, the CD8^+^ T_CM_ might be affected by ATHENA.

CD4^+^ T cells were re-clustered to define five sub-clusters. CD4^+^ T_CM_ (SELL, CCR7, LEF1, CXCR4) cells were consistent with a memory-like phenotype. CD4^+^ T_CM_ cells expressed the known effector genes (SELL, CCR7, LEF1, GZMK, CD44). CD4^+^T_PEX_ cells were enriched for SELL, TCF7, CXCR4, TIGIT, EOMES and STAT1. CD4^+^ T_EX1_ and CD4^+^ T_EX2_ highly expressed some immune checkpoint genes, such as PDCD1 or HAVCR2 (**Figures S3A, B**). In CD4^+^ T cells, we observed a significant association in which CD4^+^ T_CM_ and CD4^+^ T_CM_ cells were enriched post-treatment, while CD4^+^ T _EX2_ and CD4^+^ T _PEX_ cells were enriched in pre-treatment (**Figure S3C**). Based on trajectory analysis, the differentiation stages of CD4^+^ T_CM_ cells were more diverse (**Figures S3D, E**). The above results demonstrated that ATHENA might affect the subtype composition of T lymphocytes.

### scSTAR revealed the molecular function shift of T lymphocytes

To better understand the effect of ATHENA on the immune system, we utilized a novel algorithm named single-cell states transfer across-sample of RNA-seq data (scSTAR) (25), which analyzes cellular dynamic processes. Clustering analyses were performed on the CD8^+^ T, and CD4^+^ T populations. Each scSTAR-processed subset can be used as a cell state during treatment.

**Figure 4:**
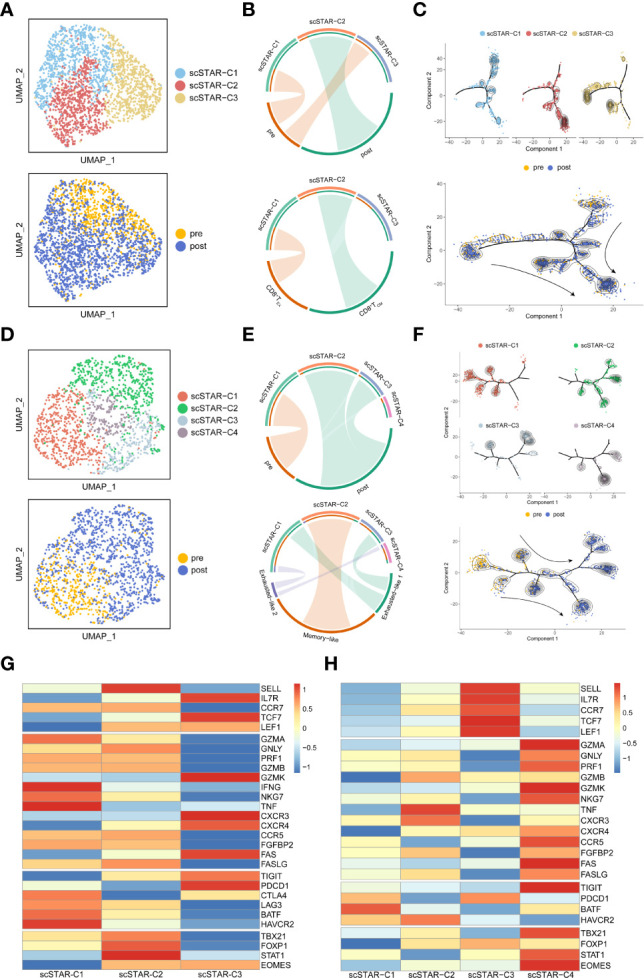
Molecular functional dynamics of CD8^+^ T cells and CD8^+^ T_CM_ cells. **(A)** UMAP of CD8^+^ T cells processed by the scSTAR algorithm, colored by sample and cluster. **(B)** The association between scSTAR clusters and treatment (above) or CD8^+^ T cell subclusters (down) in CD8^+^ T cells. The area of the ties represents the relative enrichment. **(C)** Trajectory analysis for the three scSTAR clusters of CD8^+^ T cells. Each point corresponds to an individual cell colored by cluster (above) or by sample (down). The density curves represent the distribution of each cluster. **(D)** UMAP of CD8^+^ T_CM_ cells processed by the scSTAR algorithm, colored by sample and cluster. **(E)** The association between scSTAR-processed clusters and treatment (above) or CD8^+^ T_CM_ cell subclusters (down) in CD8^+^ T_CM_ cells. The area of the ties represents the relative enrichment. **(F)** Trajectory analysis for the four scSTAR clusters of CD8^+^ T_CM_ cells. Each point corresponds to an individual cell colored by cluster (above) or by sample (down). The density curves represent the distribution of each cluster. **(G)** Heatmap of scaled normalized expression for scSTAR cell function genes in CD8^+^ T cells. **(H)** Heatmap of scaled normalized expression for scSTAR cell function genes in CD8^+^ T_CM_ cells.

To explore the association between these processed clusters and samples, we used enrichment analysis and trajectory analysis. In CD8^+^ T cells, three subsets were re-clustered after scSTAR processed (**Figure 4A**). scSTAR-C2 cells were associated with post-treatment and CD8^+^ T_CM_, while scSTAR-C1 cells were associated with pre-treatment and CD8^+^ T_EX_ (**Figure 4B**). Trajectory analysis reflected that each cluster was at the end of branches, which means a possible transition from scSTAR-C1 to scSTAR-C2 (**Figure 4C**). We also performed these analyses in CD8^+^ T_EM_. scSTAR-C2 cells and scSTAR-C3 cells were associated with post-treatment and Memory-like or Exhausted-like 1 cells, while scSTAR-C1 cells were associated with pre-treatment and Exhausted-like 1 cells or Exhausted-like 2 cells (**Figures 4D, E**). Trajectory analysis reflected that a possible transition from scSTAR-C1 to scSTAR-C2 and scSTAR-C1 to scSTAR-C2 or scSTAR-C3 (**Figure 4F**). Combining the above analysis, scSTAR-C1 of CD8^+^ T cells might differentiate toward scSTAR-C2 of CD8^+^ T cells and scSTAR-C1 of CD8^+^ T_CM_ cells might differentiate toward scSTAR-C2 of CD8^+^ T_CM_ cells or scSTAR-C3 of CD8^+^ T_CM_ cells during the treatment. Thus, we assumed that scSTAR-C2 of CD8^+^ T cells, scSTAR-C2 of CD8^+^ T_CM_ cells and scSTAR-C3 of CD8^+^ T_CM_ cells might possess the feature of elevated gene dynamics in the post-treatment sample, while scSTAR-C1 of CD8^+^ T cells and scSTAR-C1 of CD8^+^ T_CM_ cells might possess the feature of decreased gene dynamics during ATHENA treatment. Such results indicated that ATHENA treatment triggered the decreased expression of exhausted genes (PDCD1, LAG3, BATF, HAVCR2) and elevated expression of memory genes (SELL, LEF1, TCF7, TBX21, CXCR4, FASLG, FOXP1) (**Figures 4G, H**). Even though no obvious changes in CD8^+^ T_EX_ cells before and after ATHENA treatment, we tried to explore if scSTAR could reveal the hidden molecular dynamics of CD8^+^ T_EX_ cells (**Figure S2D**). Enrichment analysis showed that scSTAR-C1 of CD8^+^ T_EX_ cells were associated with pre-treatment and PEX-like 1, while scSTAR-C3 of CD8^+^ T_EX_ cells and scSTAR-C4 of CD8^+^ T_EX_ cells were associated with post-treatment and PEX-like 1 or PEX-like 2 (**Figures S2E, F**). Thus, there was little change in cell status. In CD4^+^ T cells, three subsets were re-clustered after scSTAR processed (**Figure S4A**). Trajectory analysis revealed that scSTAR-C2 cells were distributed to the post-treatment sample compared to scSTAR-C3 cells (**Figure S4B**). We discovered that scSTAR-C1 and scSTAR-C3 cells were associated with post-treatment, CD4^+^ T_CM_, CD4^+^ T_EM_ and/or CD4^+^ T_PEX_, while scSTAR-C2 cells were associated with pre-treatment, CD4^+^ T_EX1_ and/or CD4^+^ T_EX2_ (**Figure S4C**). These findings demonstrated that ATHENA might alleviate the immunosuppression of CD8^+^ T_CM_ and reverse the exhaustion state of CD4^+^ T cells to the memory phenotype during treatment.

### scSTAR analysis revealed that ATHENA activated the anti-tumoral functions of T cells

**Figure 5:**
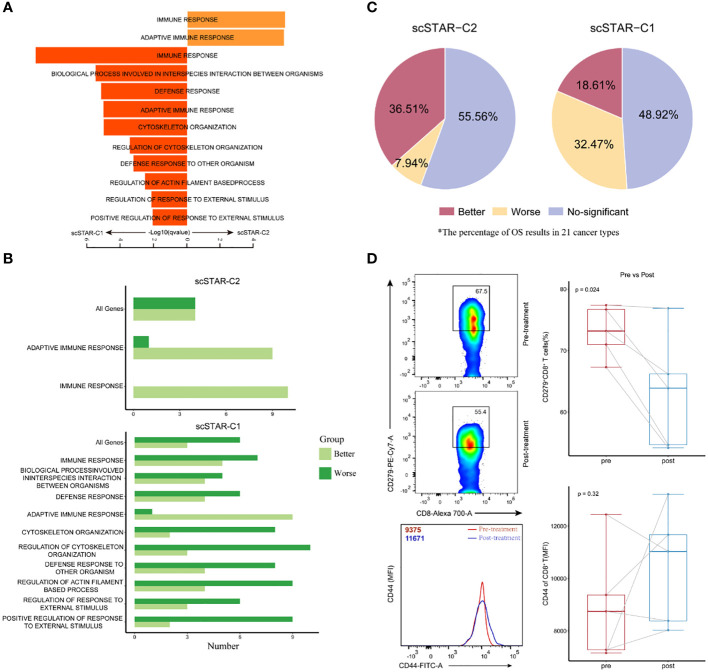
Molecular characteristics of CD8^+^ T after treatment is associated with improved overall survival. **(A)** Pathway enrichment analysis of genes in scSTAR-C1 and scSTAR-C2, respectively. The bar plot showed the top 10 enriched GO pathways. Benjamini-Hochberg (BH) adjusted p value < 0.05. **(B)** The number of associations between all changed genes, pathways in which scSTAR-C1 or scSTAR -C2 was involved and better or worse overall survival in the 21 tumor types. **(C)** The percentage of prognosis results predicted by scSTAR-C2 and scSTAR-C3 in 21 cancer types. **(D)** Flow cytometry analysis of the frequency of CD279^+^CD8^+^ T cells (upper) and the quantification of CD44 protein level (below) for 5 paired samples, pre-gated on live/CD3+ T cells and live/CD3+/CD8+ T cells.

By comparing the transcriptomic dynamics of each scSTAR-processed cluster in CD8^+^ T cells, we obtained 87 highly expressed genes in scSTAR-C2 and 100 highly expressed genes in scSTAR-C1 (**Supplementary Table 1**). This result suggested that the expression of the 87 genes was upregulated, while the expression of the other 100 genes was downregulated after ATHENA treatment. GSEA (19, 20) identified a few GO terms enriched in the above DEGs. The 87 scSTAR-C2 marker genes upregulated after treatment, including TBX21, CD8A, FASLG, KLRC4, SLAMF7, IGKV families and TRBV families, were involved in the adaptive immune response and immune response pathways. In contrast, the 100 scSTAR-C1 marker genes downregulated after treatment, including HAVCR2, LAG3, PRDM1, BATF, PDGFRB and TNFRSF1A, were involved in 10 pathways, such as regulation of response to external stimulus, defense response and cytoskeleton organization (**Figure 5A** and **Supplementary Table 2**).

The differences between scSTAR-C1 and scSTAR-C2 cells with respect to pathway enrichment prompted us to investigate their role in ATHENA treatment. To address the challenge of not being able to assess the efficacy of ATHENA in most tumors due to the scarcity of cases, we put the genes involved in pathways into Kaplan-Meier for pan-cancer (21 cancer types) (22), which indirectly evaluated the therapeutic ability of ATHENA. In 21 cancer types, 36.51% of cancers had significantly better OS predicted by the 87 scSTAR-C2 marker genes upregulated after ATHENA treatment, while only 18.61% of cancers had significantly better OS predicted by the 100 scSTAR-C1 marker genes downregulated after ATHENA treatment. Additionally, 7.94% of cancers had significantly worse OS predicted by the 87 scSTAR-C2 marker genes upregulated after treatment, while 32.47% of cancers had significantly worse OS predicted by the 100 scSTAR-C1 marker genes downregulated after treatment (**Figures 5B, C**). The results demonstrated an association between upregulated genes after treatment and the percentage of significantly improved OS in the 21 tumor types. This suggested that ATHENA may activate anti-tumoral immunity by upregulating the genes involved in the adaptive immune response and immune response pathways and downregulating the genes involved in the 10 pathways. Similarly, we obtained the 299 scSTAR-C3 marker genes and the 61 scSTAR-C2 marker genes in CD4^+^ T cells (**Supplementary Table 1**). GSEA and survival analysis revealed that the 299 scSTAR-C3 marker genes favored a greater number of better OS and a smaller number of worse OS compared with the 61 scSTAR-C2 marker genes downregulated after treatment (**Figures S4A–C** and **Supplementary Table 3**).

To confirm these transcriptional findings at the protein level, we validated the status transitions of CD8^+^ T cells on paired samples derived from five patients treated with ATHENA by flow cytometry assay using the CD44 and CD279 surface markers. Comparison of matched pre- and post-treatment specimens demonstrated that the proportion of CD279^+^CD8^+^ T cells was reduced (paired-Student’s t test p=0.024) and the expression of CD44 molecule was upregulated (paired-Student’s t test p=0.32) after ATHENA treatment (**Figure 5D**). Although the change of CD44 molecule was no significant, the expression has an upward trend. These results suggested that ATHENA may reverse the exhausted status of CD8^+^ T cells. Taken together, we assumed that ATHENA might improve the activity of T cells to induce tumor regression by reducing exhausted status.

### Fine clustering of B and myeloid cells

**Figure 6:**
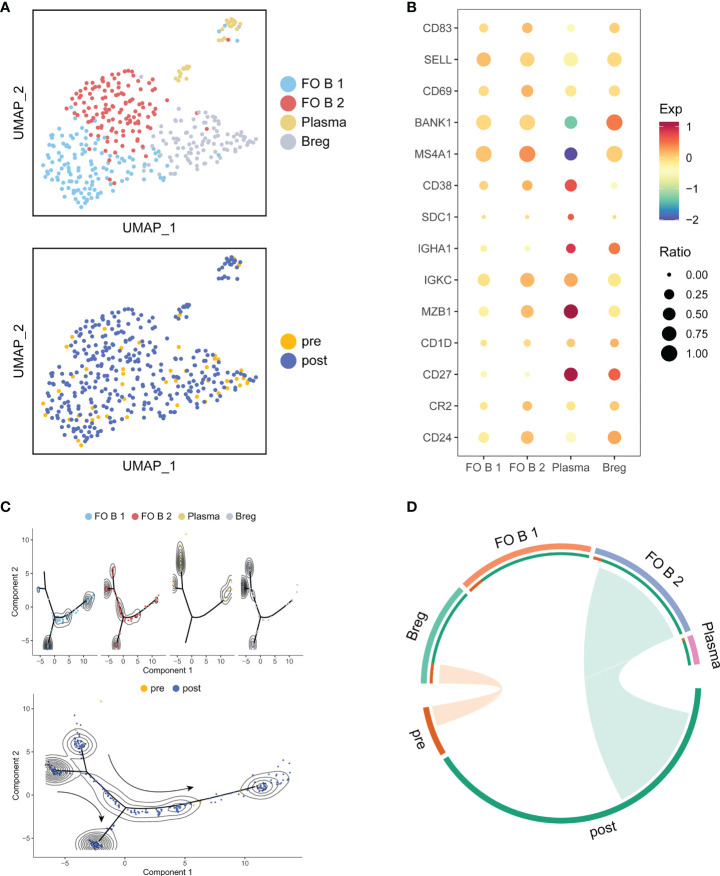
Detailed characterization of B cells and their association with treatment. **(A)** UMAP of B cells, colored by cell type and sample. **(B)** Bubble plot of selected B cells function-associated genes in each cell cluster. **(C)** Pseudotime analysis of B cells inferred by Monocle2. Each point corresponds to an individual cell colored by cluster (above) or by treatment (down). The density curves represent the distribution of each cluster. **(D)** The association between subtypes and treatment in B cells. The area of the ties represents the relative enrichment.

Since B and Myeloid cells had a larger change in cell proportions after treatment, we also identified four sub-clusters in B cells and Myeloid cells, respectively. Follicular B 1 (FO B 1) and Follicular B 2 (FO B 2) cells highly expressed CD83, CD69, SELL, and BANK1 (26). Plasma cells were enriched for CD38, SDC1, IGHA1, IGKC and MZB1 (27, 28). Regulatory B cells (Bregs) were enriched for CD27, CD24, CD1D and CR2, which were a new subset of B cells with immunosuppressive functions resembling those of T cells (29) (**Figures 6A, B**). Compared to pre-treatment, trajectory analysis revealed that the differentiation of Breg cells and Plasma cells toward FO B 1 cells after treatment (**Figure 6C**). We also observed that Bregs were associated with pre-treatment, but FO B 2 cells were associated with post-treatment (**Figure 6D**). Myeloid cells were composed of CD14^+^ Mono cells, CD16^+^ Mono cells, M1 cells and M1/M2 cells (**Figures S5A, B**). Trajectory analysis did not show a possible state change between these sub-clusters (**Figure S5C**). Only CD16^+^ Mono cells were associated with post-treatment (**Figure S5D**). We assumed that ATHENA also might affect the subtype composition of B and Myeloid cells.

### ATHENA also may enhance the anti-tumoral effect of B cells

**Figure 7:**
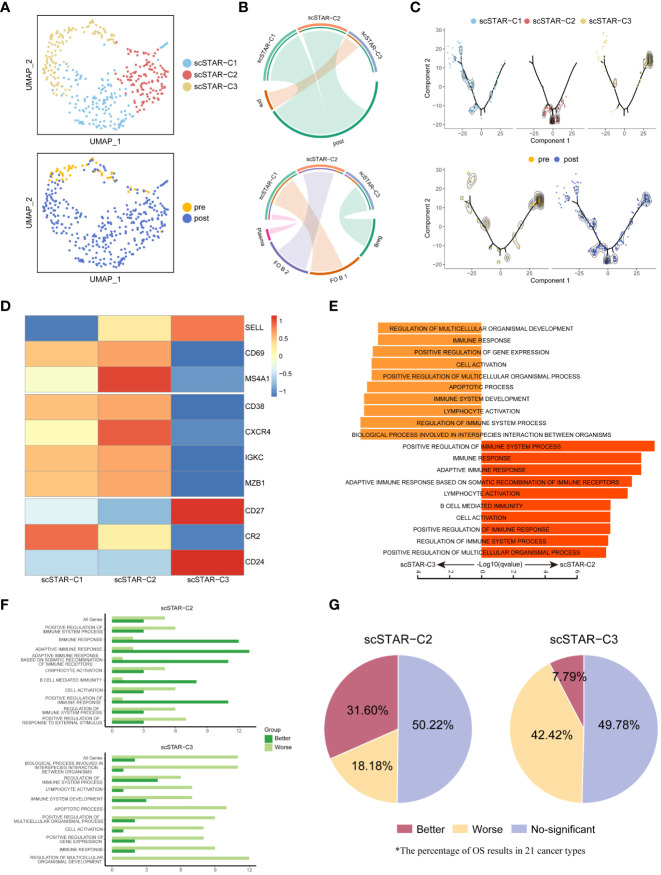
The association between molecular functional dynamics of B cells subsets and prognosis. **(A)** UMAP of B cells processed by the scSTAR algorithm, colored by sample and cluster. **(B)** The association between scSTAR-processed clusters and treatment (above) or B cell subclusters (down). The area of the ties represents the relative enrichment. **(C)** Trajectory analysis for the three scSTAR-processed clusters. Each point corresponds to an individual cell colored by cluster (above) or by sample (down). The density curves represent the distribution of each cluster. **(D)** Heatmap of scaled normalized expression for B cell function genes. **(E)** Pathway enrichment analysis of genes in scSTAR-C2 and scSTAR-C3, respectively. The bar plot showed the top 10 enriched GO pathways. Benjamini-Hochberg (BH) adjusted p value < 0.05. **(F)** The number of associations between all changed genes, pathways in which scSTAR-C2 or scSTAR-C3 was involved and better or worse overall survival in the 21 tumor types. **(G)** The percentage of prognosis results predicted by scSTAR-C2 and scSTAR-C3 in 21 cancer types.

In B cells, three subsets were re-clustered after scSTAR processed (**Figure 7A**). scSTAR-C1 and scSTAR-C2 were associated with post-treatment, plasma, FO B 1 and FO B 2, while scSTAR-C3 cells were associated with pre-treatment and Breg (**Figure 7B**). Trajectory analysis reflected that scSTAR-C2 and scSTAR-C3 were at the end of branches, which means a possible transition from scSTAR-C3 to scSTAR-C2 (**Figure 7C**). We observed that scSTAR-C2 highly expressed plasma-associated genes (CD38, CXCR4, IGKC and MZB1), while scSTAR-C3 highly expressed Breg-associated genes (CD27, CD24) (**Figure 7D**). We deduced that scSTAR-C3 might differentiate toward scSTAR-C2 during the treatment. Therefore, we hypothesized that scSTAR-C2 might dynamically possess the feature of elevated plasma-associated genes during treatment, while scSTAR-C3 might dynamically possess the feature of decreased Breg-associated gene. In Myeloid cells, three subsets were re-clustered after scSTAR processed (**Figure S6A**). scSTAR-C3 was associated with post-treatment and CD16^+^ Mono, while scSTAR-C2 was associated with pre-treatment, M1/M2 and M2. (**Figures S6A, B**). Cell states in the trajectory revealed that a possible transition from scSTAR-C2 to scSTAR-C3 (**Figure S6C**). We also observed that scSTAR-C3 highly expressed CD16^+^ Mono-associated genes (FCGR3A, CX3CR1), while scSTAR-C2 highly expressed M2-associated genes (CD68, CD163, CLEC7A, CTSD, MP9, TNFSF8) (**Figure S6D**). Thus, scSTAR-C2 might differentiate toward scSTAR-C3 during the treatment. We hypothesize that scSTAR-C3 may be dynamically characterized by elevated CD16^+^Mono-associated genes during treatment, while scSTAR-C2 may be dynamically characterized by decreased M2-associated genes.

For further understood the efficacy of ATHENA, we obtained 91 scSTAR-C2 marker genes, 70 scSTAR-C3 marker genes in B cells and 475 scSTAR-C3 marker genes, 163 scSTAR-C2 marker genes in Myeloid cells (**Supplementary Table 1**). As with the above analyses, A few GO terms were enriched in the above marker genes by GSEA analysis. In B cells, the 91 scSTAR-C2 marker genes upregulated after treatment were involved in top-ranked 10 pathways, such as adaptive immune response, B cell mediated immunity and lymphocyte activation (including CD40, IL4R, IGHD, GAPT and HLA families). In contrast, the 70 scSTAR-C3 genes downregulated after treatment were involved in top-ranked 10 pathways, such as apoptotic process and immune system development (including CD24, CD27, FOS and ITGB1) (**Figure 7E** and **Supplementary Table 4**). In Myeloid cells, the 475 scSTAR-C3 marker genes upregulated after treatment were involved in top-ranked 10 pathways, such as leukocyte differentiation and cell activation (including IL1B, CD86, BATF3 and LYN). In contrast, the 163 scSTAR-C2 marker genes downregulated after treatment were involved in top-ranked 10 pathways, such as defense response and innate immune response (including JUND, CD68, HIF1A and CD14) (**Figure S6D** and **Supplementary Table 5**). Then we put the genes involved in pathways into Kaplan-Meier for pan-cancer (21 cancer types). In B cells, 31.6% of cancers had significantly better OS predicted by the 91 scSTAR-C2 marker genes upregulated after ATHENA treatment, while only 7.79% of cancers had significantly better OS predicted by the 70 scSTAR-C3 marker genes downregulated after ATHENA treatment. 18.18% of cancers had significantly worse OS predicted by the 91 scSTAR-C2 marker genes upregulated after treatment, while 42.42% of cancers had significantly worse OS predicted by the 70 scSTAR-C3 marker genes downregulated after ATHENA treatment (**Figures 7F, G**). In Myeloid cells, 12.12% of cancers had significantly better OS predicted by the 475 scSTAR-C3 marker genes upregulated after ATHENA treatment, while 21.65% of cancers had significantly better OS predicted by the 163 scSTAR-C2 marker genes downregulated after ATHENA treatment. 27.27% of cancers had significantly worse OS predicted by the 475 scSTAR-C3 marker genes upregulated after treatment, while 24.24% of cancers had significantly worse OS predicted by the 163 scSTAR-C2 marker genes downregulated after ATHENA treatment (**Figures S6F, G**). There was no obvious change in OS before and after treatment. Thus, ATHENA might transform the status of the Myeloid cells, but did not improve the prognosis of the tumor. Based on the above analyses, B cells had a significant association between upregulated genes after treatment and the percentage of significantly improved OS in the 21 tumor types. This suggested that ATHENA may exert anti-tumor effects by alleviating the immunosuppression of Bregs on T cells and activating the effects of B cells.

### Transcription factors were driven by ATHENA in T cells

Given the dramatic transitions of cell subtypes in CD8^+^ T cells, we hypothesized that some transcription factors (TFs) might serve as master regulators that enhance CD8^+^ T cell activity. Therefore, CD8^+^ T cells were applied to SCENIC (23) to reconstruct the gene regulatory network (GRN). The output was an activity score matrix of regulons per cell, which represents the link between transcription factors and their direct target gene.

**Figure 8:**
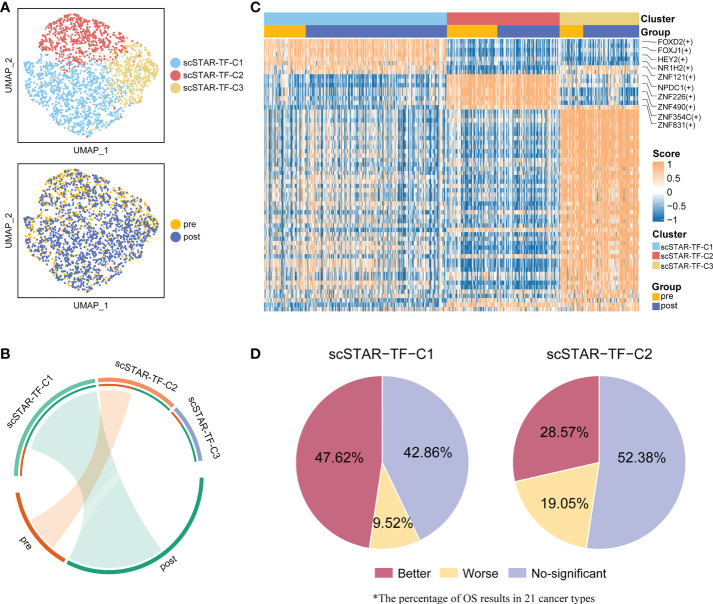
scSTAR analysis of gene regulatory networks in CD8^+^ T cells. **(A)** UMAP of CD8^+^ T cells processed by the scSTAR algorithm and SCENIC, colored by sample and cluster. **(B)** The association between scSTAR-processed clusters and treatment (left) or CD8^+^ T cell sub-clusters (right). The area of the ties represents the relative enrichment. **(C)** Heatmap of scaled normalized regulon activity for CD8^+^ T cells as determined by two-sided Wilcoxon rank-sum test with Bonferroni FDR correction (q < 0.05). **(D)** The percentage of results predicted by scSTAR-TF-C1 and scSTAR-TF-C2 in 21 cancer types.

To better reveal the change in regulon activity before and after treatment, scSTAR was also performed on the regulon matrix. In CD8^+^ T cells, we obtained three clusters and their differentially activated regulons (DRAs). Enrichment analysis showed that scSTAR-TF-C1 was associated with post-treatment and scSTAR-TF-C2 was associated with pre-treatment. Because scSTAR-TF-C3 had no association with pre- and post-treatment, we inferred that it mixed cells from pre- and post-treatment states and might be in a state of transition (**Figures 8A, B**). To further assess the role of regulons in prognosis, pan-cancer Kaplan–Meier survival analyses were performed on transcription factor activity. The top-ranked five TFs were selected, including FOXD2, FOXJ1, NR1H2, ZNF121, and HEY2 for scSTAR-TF-C1 and NPDC1, ZNF226, ZNF490, ZNF831, and ZNF354C for scSTAR-TF-C2 (**Figure 8C**). A total of 47.62% of cancers that highly expressed the top-ranked five TFs of scSTAR-TF-C1 had significantly better OS, while only 28.57% of cancers had significantly better OS predicted by the top-ranked five TFs of scSTAR-TF-C2. Additionally, only 9.52% of cancers that highly expressed the top-ranked five TFs of scSTAR-TF-C1 had significantly worse OS, while 19.05% of cancers had significantly worse OS predicted by the top-ranked five TFs of scSTAR-TF-C2 (**Figure 8D**). The results demonstrated that high regulon activities of scSTAR-TF-C1 favored a higher percentage of improved prognosis, while high regulon activities of scSTAR-TF-C2 favored a higher percentage of worse prognosis.

A similar phenomenon was observed in CD4^+^ T cells. There were five clusters and the DRAs of each cluster (**Figure S7A**). Among these clusters, scSTAR-TF-C1 and scSTAR-TF-C4 were associated with post-treatment, and scSTAR-TF-C2, scSTAR-TF-C3 and scSTAR-TF-C5 were associated with pre-treatment (**Figure S7B**). As expected, the DRAs of scSTAR-TF-C1 could predict a higher percentage of better prognosis than the DRAs of scSTAR-TF-C2, scSTAR-TF-C3 and scSTAR-TF-C5 (**Figures S7C, D**). We also performed the above analyses on B cells. Three clusters and DRAs of each cluster were identified (**Figures S8A, B**). scSTAR-TF-C2 was associated with pre-treatment and scSTAR-TF-C3 was associated with post-treatment (**Figure S8B**). In 21 cancer types, we failed to observe a change in OS for these DRA before and after treatment (**Figure S8D**). Thus, we inferred that these top-ranked five DRAs of CD8^+^ or CD4^+^ T cells for each cluster associated with post-treatment might be driven by ATHENA to improve prognosis.

## Discussion

Our previous studies showed that irradiated cancer cell vaccines enhance anti-tumor efficacy by stimulating an intensive T-cell response in a mouse model (11, 17). Thus, we developed an improved whole-cell tumor vaccines called ATHENA. In this study, we performed the ATHENA technique on a 6-year-old patient diagnosed with meningeal rhabdomyosarcoma. After treatment, the patient achieved CR due to the regression in most lesions. Here, we leveraged deep scRNA-seq to explore the dynamics of PBMCs before and after treatment. With the scSTAR algorithm, an exciting phenomenon that the exhausted status was shifted towards memory status in T cells and Breg cells were converted to Plasma or Follicular B cells during ATHENA treatment was observed. Survival analysis and transcription factor analysis revealed enhancement of T cell activity and anti-tumor effects. Through this study, we provide a proven approach for exploring the mechanisms of rare cases or personalized cancer treatment and the introduction of ATHENA may be an alternative complement for cancer immunotherapy.

Although there was little change in T cells regarding cell proportions, a variety of cell states were likely to occur. With the scSTAR algorithm, we revealed the molecular dynamics features of CD8^+^ T and CD4^+^ T cells during treatment. We discovered that memory-like genes (SELL, LEF1, CCR7, TBX21, CXCR4, FASLG, FOXP1) were upregulated, while exhausted genes (HAVCR2, PDCD1, CTLA4, BATF, LAG3) were downregulated after ATHENA treatment.

Based on the larger change in the proportion of B and Myeloid cells before and after treatment, we also explored the molecular dynamics features of B and Myeloid cells during treatment. The marker genes of Follicular B or Plasma cells were upregulated, while the marker genes of Bregs were downregulated after ATHENA treatment. Therefore, we assumed that ATHENA may not only alleviate the immunosuppression effect of Bregs on T cells and enhance the anti-tumoral activity of Follicular B and Plasma cells, but regain the differentiation and effector potential of T cells by reversing the exhausted phenotype.

Given the scarcity of cases and the difficulty in biopsies, accurate assessment of efficacy poses a challenge to our study. We infer that peripheral circulating immunity reflects to some extent the prognosis of the disease. Putting the 87 scSTAR-C2 marker genes and the 100 scSTAR-C1 marker genes in CD8^+^ T cells, the 299 scSTAR-C3 marker genes and the 61 scSTAR-C2 marker genes in CD4^+^ T cells, the 91 scSTAR-C2 marker genes and the 70 scSTAR-C3 marker genes in B cells into Kaplan-Meier survival analysis for pan-cancer revealed that ATHENA not only increased the proportion of superior prognosis but also decreased the proportion of inferior prognosis. We further demonstrate that transcription factors with enhanced activity in T cells after treatment predict a greater proportion of better prognosis as well. Therefore, T cell transitions with enhanced activity trigger systemic peripheral immunity to kill cancer cells.

Current immunotherapies, such as ICB or CAR-T therapy, are approximately 20% effective in the overall population (30). According to the results of survival analysis for 21 cancer types, ATHENA may have unexpectedly high efficiency beyond current immunotherapies. Previous studies reported that PD-1 inhibitors could reverse the exhausted state of terminally exhausted Texp cells during chronic viral infection (20, 31). This result was similar with our results. Therefore, the combination of anti-PD-1 inhibitors and ATHENA may have a synergistic potential to increase the efficiency of immunotherapy. Despite an initial glimpse into the molecular dynamics of PBMCs before and after ATHENA treatment from a single-cell perspective, we still need more cases, samples and experiments to refine our studies. More time is needed for follow-up to understand the clinical efficacy and adverse effects.

Here, our central work introduces the clinical outcome of one case treated with ATHENA and its potential dynamic changes in immune molecules before and after treatment. Based on our study, we also aimed to present a unique approach to analyze the feature of rare cases or the mechanism of personalized treatment. More trials were needed to identify tumors that are sensitive to ATHENA and explore effective prediction biomarkers. In addition, combining ATHENA with nanoparticle systems or ICIs may be a promising method to enhance the efficacy of treatment. Therefore, our current research paves the way for the dissemination of ATHENA technology and unique analytical methods.

## Data availability statement

The original contributions presented in the study are included in the article/**Supplementary Materials**. Further inquiries can be directed to the corresponding authors.

## Ethics statement

The studies involving human participants were reviewed and approved by The Ethics Committee of the Jinshan Hospital of Fudan University (Approved ID: JIEC-2019-09). Written informed consent to participate in this study was provided by the participants’ legal guardian/next of kin. Written informed consent was obtained from the individual(s), and minor(s)’ legal guardian/next of kin, for the publication of any potentially identifiable images or data included in this article.

## Author contributions

YL and KW conducted most of the study and analyzed the data; KW prepared the tumor vaccines, collected PBMC samples and performed the flow cytometry. YL analyzed the data, wrote the manuscript and performed the flow cytometry. XZ and TQ conceived, designed, and supervised the study. YZ, XBZ, SS, and FQ provided instructions and another help. All authors contributed to the article and approved the submitted version.

## Funding

This work was supported by the Key Subject Construction Program of Shanghai Health Administrative Authority (Grant No. ZK2019B30) and the Jinshan Science and Technology Commission (Grant Number: 2018-3-5).

## Acknowledgments

We thank Jingyao Chen, Furong Yan, Zhicheng Yang, Ying Yang, and Xiaoping He for their help with this study. The first author wants to thank Zijun Zhai for her support and help in his life and work.

## Conflict of interest

The authors declare that the research was conducted in the absence of any commercial or financial relationships that could be construed as a potential conflict of interest.

## Publisher’s note

All claims expressed in this article are solely those of the authors and do not necessarily represent those of their affiliated organizations, or those of the publisher, the editors and the reviewers. Any product that may be evaluated in this article, or claim that may be made by its manufacturer, is not guaranteed or endorsed by the publisher.

## Supplementary material

Supplementary Figure 1 Trajectory analysis of CD8^+^ T cells. **(A)** Pseudotime analysis of CD8^+^ T cells inferred by Monocle2. Each point corresponds to an individual cell colored by cluster The density curves represent the distribution of each cluster. **(B)** Corresponding to Figure S1A. Each point corresponds to an individual cell colored by cluster The density curves represent the distribution of treatment.

Supplementary Figure 2 Detailed characterization of CD8^+^ T_EX_ cells and molecular functional dynamics of CD8^+^ T cell subsets. **(A)** UMAP of all CD8^+^ T_Ex_ cells, colored by cell type and sample. **(B)** Bubble plot of selected CD8^+^ T_Ex_ cell function-associated genes in each cell cluster. **(C)** Pseudotime analysis of CD8^+^ T_EX_ cells inferred by Monocle2. Each point corresponds to an individual cell colored by cluster (left) or by treatment (right). The density curves represent the distribution of each cluster. **(D)** UMAP of CD8^+^ T_EX_ cells processed by the scSTAR algorithm, colored by sample and cluster. **(B)** The association between scSTAR-processed clusters and treatment (above) or CD8^+^ T_EX_ subclusters (down). The area of the ties represents the relative enrichment. **(F)** Heatmap of scaled normalized expression for scSTAR-cluster function genes.

Supplementary Figure 3 Detailed characterization of CD4^+^ T cells and their association with treatment. **(A)** UMAP of all CD4^+^ T cells, colored and labeled by cell type. **(B)** Heatmap of selected CD4^+^ T cell function-associated genes in each cell cluster. **(C)** The association between subtypes and treatment. The area of the ties represents the relative enrichment. **(D)** Pseudotime analysis of CD4^+^ T cells inferred by Monocle2. Each point corresponds to an individual cell colored by treatment. The density curves represent the distribution of each cluster. **(E)** Changes in CD4T trajectory before and after treatment. Each point corresponds to an individual cell colored by treatment. The density curves represent the distribution of each cluster.

Supplementary Figure 4 The association between molecular functional dynamics of CD4^+^ T cell subsets and prognosis. **(A)** UMAP of CD4^+^ T cells processed by the scSTAR algorithm, colored by sample and cluster. **(B)** Trajectory analysis for the three scSTAR-processed clusters. Each point corresponds to an individual cell colored by cluster (above) or by sample (down). The density curves represent the distribution of each cluster. **(C)** The association between scSTAR-processed clusters and treatment (above) or CD4^+^ T cell subclusters (down). The area of the ties represents the relative enrichment. **(D)** Pathway enrichment analysis of genes in in scSTAR-C2 and scSTAR-C3, respectively. The bar plot showed the top 10 enriched GO pathways. Benjamini-Hochberg (BH) adjusted p value < 0.05. **(E)** The number of associations between all changed genes, pathways in which scSTAR-C2 or scSTAR-C3 was involved and better or worse overall survival in the 21 tumor types. **(F)** The percentage of prognosis results predicted by scSTAR-C2 and scSTAR-C3 in 21 cancer types.

Supplementary Figure 5 Detailed characterization of Myeloid cells and their association with treatment. **(A)** UMAP of all Myeloid cells, colored by cell type and sample. **(B)** Bubble plot of selected Myeloid cells function-associated genes in each cell cluster. **(C)** Pseudotime analysis of Myeloid cells derived from PBMC samples inferred by Monocle2. Each point corresponds to an individual cell colored by cluster (above) or by treatment (down). The density curves represent the distribution of each cluster. **(D)** The association between subtypes and treatment in Myeloid cells. The area of the ties represents the relative enrichment.

Supplementary Figure 6 The association between molecular functional dynamics of Myeloid cell subsets and prognosis. **(A)** UMAP of Myeloid cells processed by the scSTAR algorithm, colored by sample and cluster. **(B)** The association between scSTAR-processed clusters and treatment. The area of the ties represents the relative enrichment. **(C)** Trajectory analysis for the three scSTAR-processed clusters. Each point corresponds to an individual cell colored by cluster (above) or by sample (down). The density curves represent the distribution of each cluster. **(D)** Heatmap of scaled normalized expression for scSTAR cell function genes. **(E)** Pathways enriched in Myeloid cells in scSTAR-C2 and p Myeloid-C3. The bar plot showed the top 10 enriched GO pathways. Benjamini-Hochberg (BH) adjusted p value < 0.05. **(F)** The number of associations between all changed genes, pathways in which scSTAR-C2 or scSTAR-C3 was involved and better or worse overall survival in the 21 tumor types. **(G)** The percentage of prognosis results predicted by scSTAR-C2 and scSTAR-C3 in 21 cancer types.

Supplementary Figure 7 scSTAR analysis of gene regulatory networks in CD4^+^ T cells. **(A)** UMAP of CD4^+^ T cells processed by the scSTAR algorithm and SCENIC, colored by sample and cluster. **(B)** The association between scSTAR-processed clusters and treatment. The area of the ties represents the relative enrichment. **(C)** Heatmap of scaled normalized regulon activity for CD4^+^ T cells as determined by two-sided Wilcoxon rank-sum test with Bonferroni FDR correction (q < 0.05). **(D)** The percentage of prognosis results predicted by scSTAR-processed clusters in 21 cancer types.

Supplementary Figure 8 scSTAR analysis of gene regulatory networks in B cells. **(A)** UMAP of B cells processed by the scSTAR algorithm and SCENIC, colored by sample and cluster. **(B)** The association between scSTAR-processed clusters and treatment. The area of the ties represents the relative enrichment. **(C)** Heatmap of scaled normalized regulon activity for B cells as determined by two-sided Wilcoxon rank-sum test with Bonferroni FDR correction (q < 0.05). **(D)** The percentage of prognosis results predicted by scSTAR-processed clusters in 21 cancer types.
