# Supplementary figures and images for: Single-cell analyses reveal the therapeutic effects of ATHENA and its mechanism in a rhabdomyosarcoma patient
Source: Front Oncol. 2022 Nov 29;12:1039145. doi: 10.3389/fonc.2022.1039145 (PMC9745782; doi:10.3389/fonc.2022.1039145)

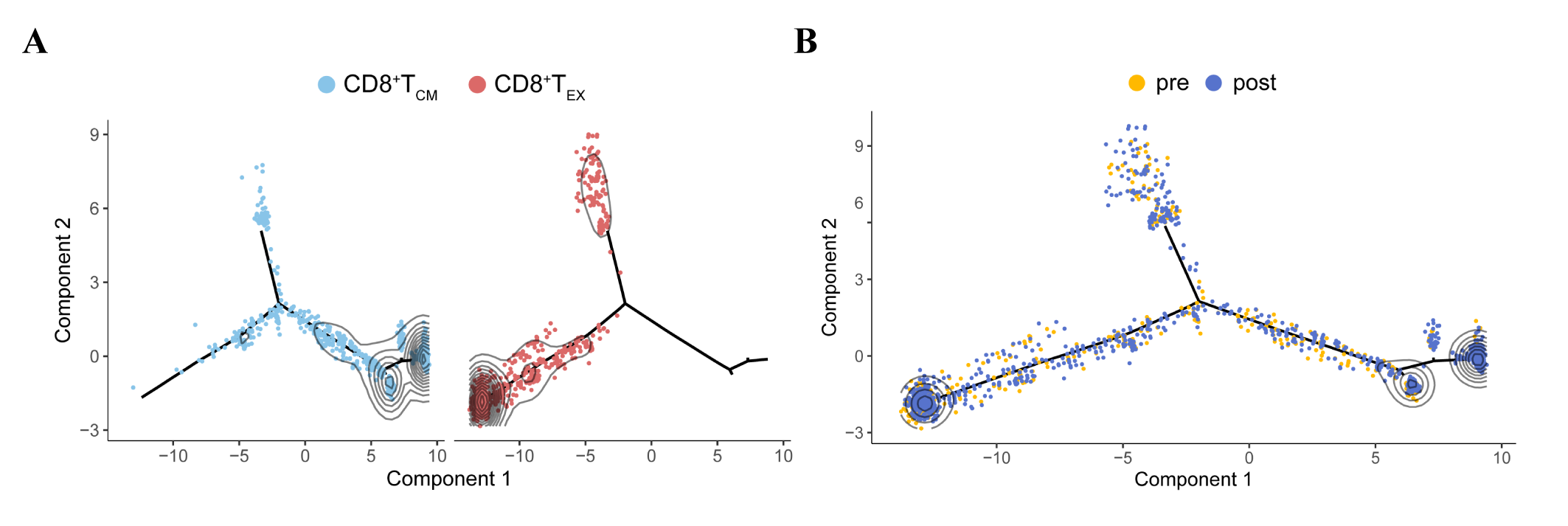

Supplement: Supplementary Figure 1 — Trajectory analysis of CD8+ T cells. (A) Pseudotime analysis of CD8+ T cells inferred by Monocle2. Each point corresponds to an individual cell colored by cluster The density curves represent the distribution of each cluster. (B) Corresponding to Figure S1A. Each point corresponds to an individual cell colored by cluster The density curves represent the distribution of treatment. [file Image_1.tif]

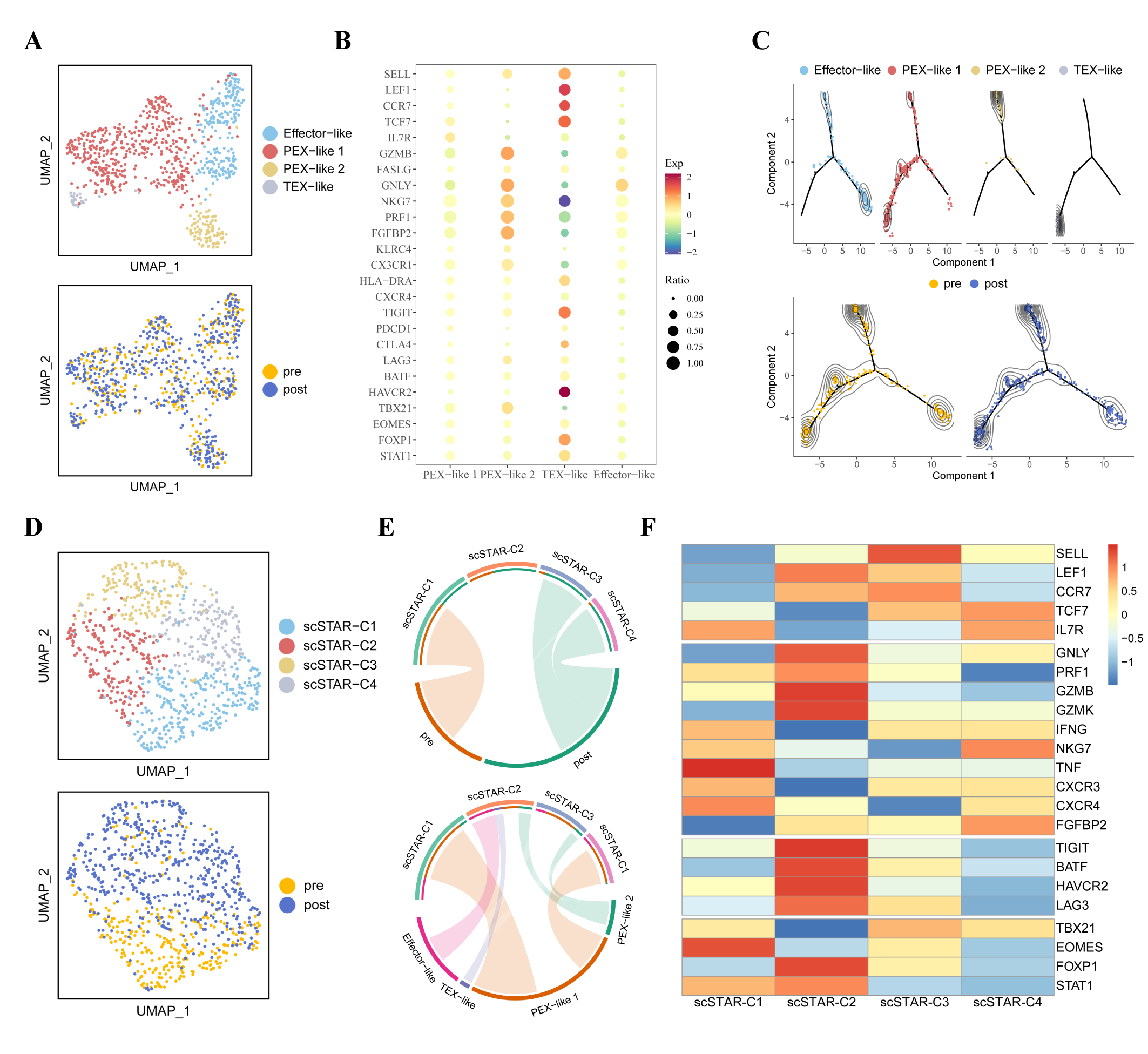

Supplement: Supplementary Figure 2 — Detailed characterization of CD8+ TEX cells and molecular functional dynamics of CD8+ T cell subsets. (A) UMAP of all CD8+ TEx cells, colored by cell type and sample. (B) Bubble plot of selected CD8+ TEx cell function-associated genes in each cell cluster. (C) Pseudotime analysis of CD8+ TEX cells inferred by Monocle2. Each point corresponds to an individual cell colored by cluster (left) or by treatment (right). The density curves represent the distribution of each cluster. (D) UMAP of CD8+ TEX cells processed by the scSTAR algorithm, colored by sample and cluster. (B) The association between scSTAR-processed clusters and treatment (above) or CD8+ TEX subclusters (down). The area of the ties represents the relative enrichment. (F) Heatmap of scaled normalized expression for scSTAR-cluster function genes. [file Image_2.tif]

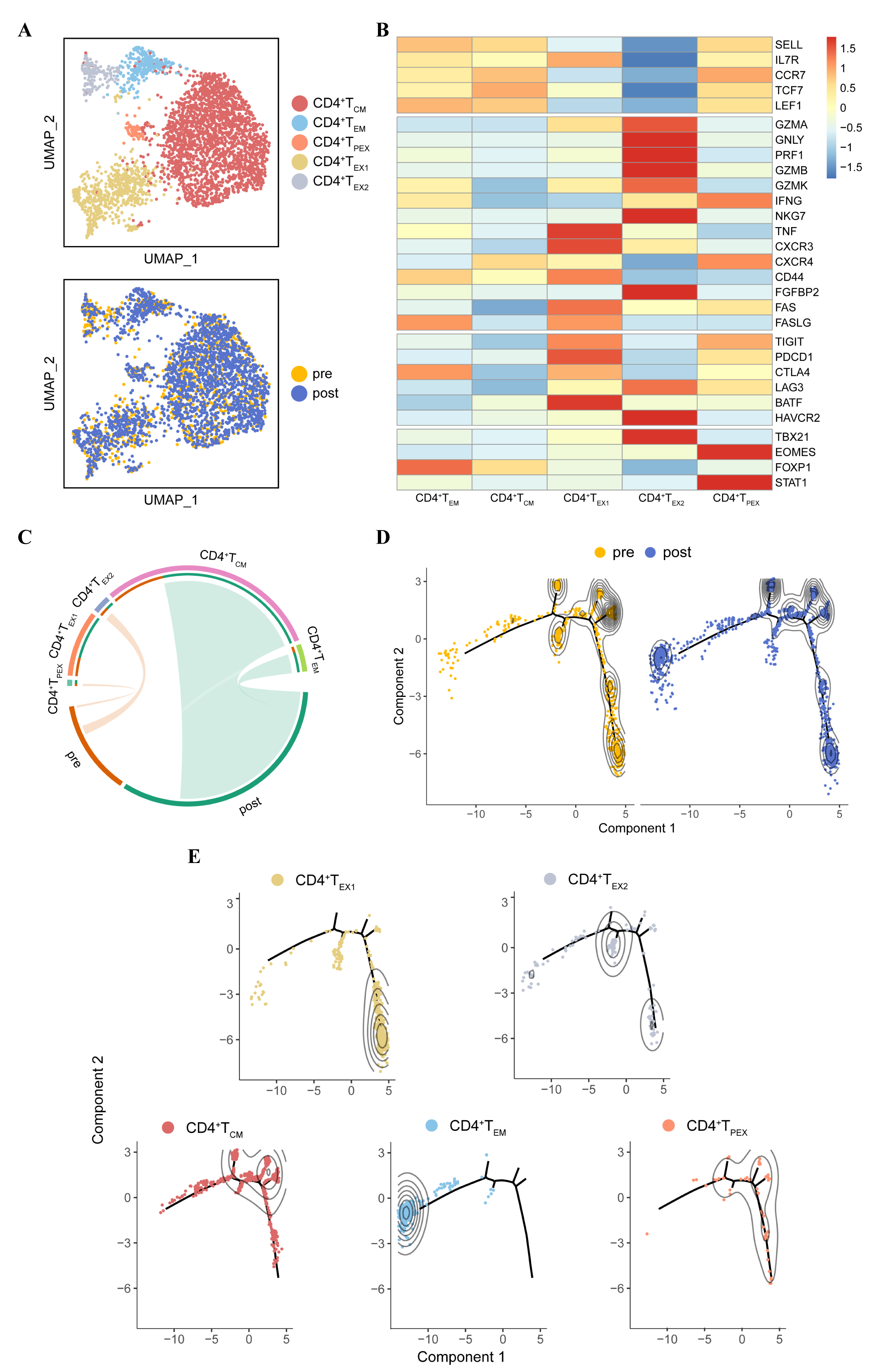

Supplement: Supplementary Figure 3 — Detailed characterization of CD4+ T cells and their association with treatment. (A) UMAP of all CD4+ T cells, colored and labeled by cell type. (B) Heatmap of selected CD4+ T cell function-associated genes in each cell cluster. (C) The association between subtypes and treatment. The area of the ties represents the relative enrichment. (D) Pseudotime analysis of CD4+ T cells inferred by Monocle2. Each point corresponds to an individual cell colored by treatment. The density curves represent the distribution of each cluster. (E) Changes in CD4T trajectory before and after treatment. Each point corresponds to an individual cell colored by treatment. The density curves represent the distribution of each cluster. [file Image_3.tif]

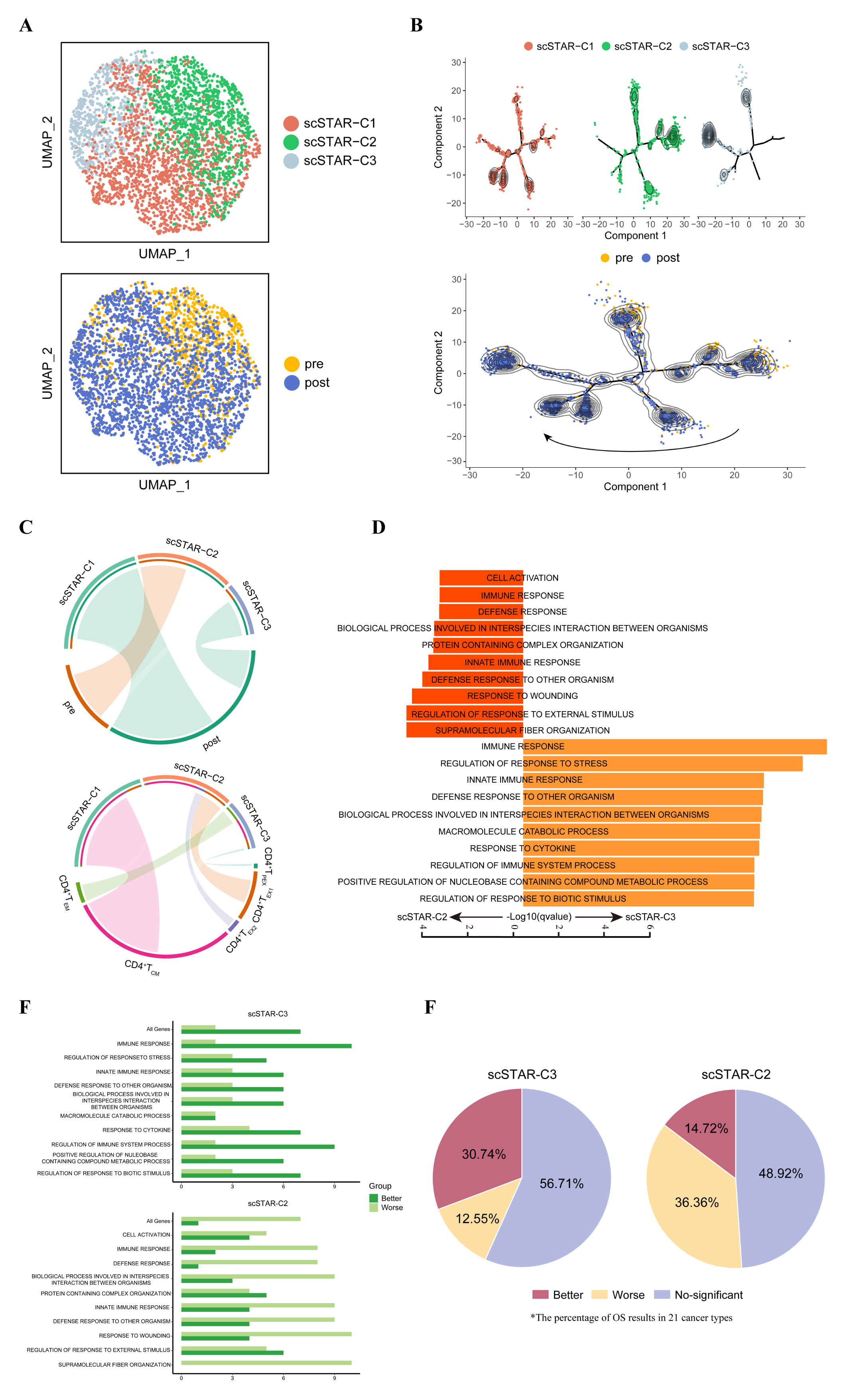

Supplement: Supplementary Figure 4 — The association between molecular functional dynamics of CD4+ T cell subsets and prognosis. (A) UMAP of CD4+ T cells processed by the scSTAR algorithm, colored by sample and cluster. (B) Trajectory analysis for the three scSTAR-processed clusters. Each point corresponds to an individual cell colored by cluster (above) or by sample (down). The density curves represent the distribution of each cluster. (C) The association between scSTAR-processed clusters and treatment (above) or CD4+ T cell subclusters (down). The area of the ties represents the relative enrichment. (D) Pathway enrichment analysis of genes in in scSTAR-C2 and scSTAR-C3, respectively. The bar plot showed the top 10 enriched GO pathways. Benjamini-Hochberg (BH) adjusted p value < 0.05. (E) The number of associations between all changed genes, pathways in which scSTAR-C2 or scSTAR-C3 was involved and better or worse overall survival in the 21 tumor types. (F) The percentage of prognosis results predicted by scSTAR-C2 and scSTAR-C3 in 21 cancer types. [file Image_4.tif]

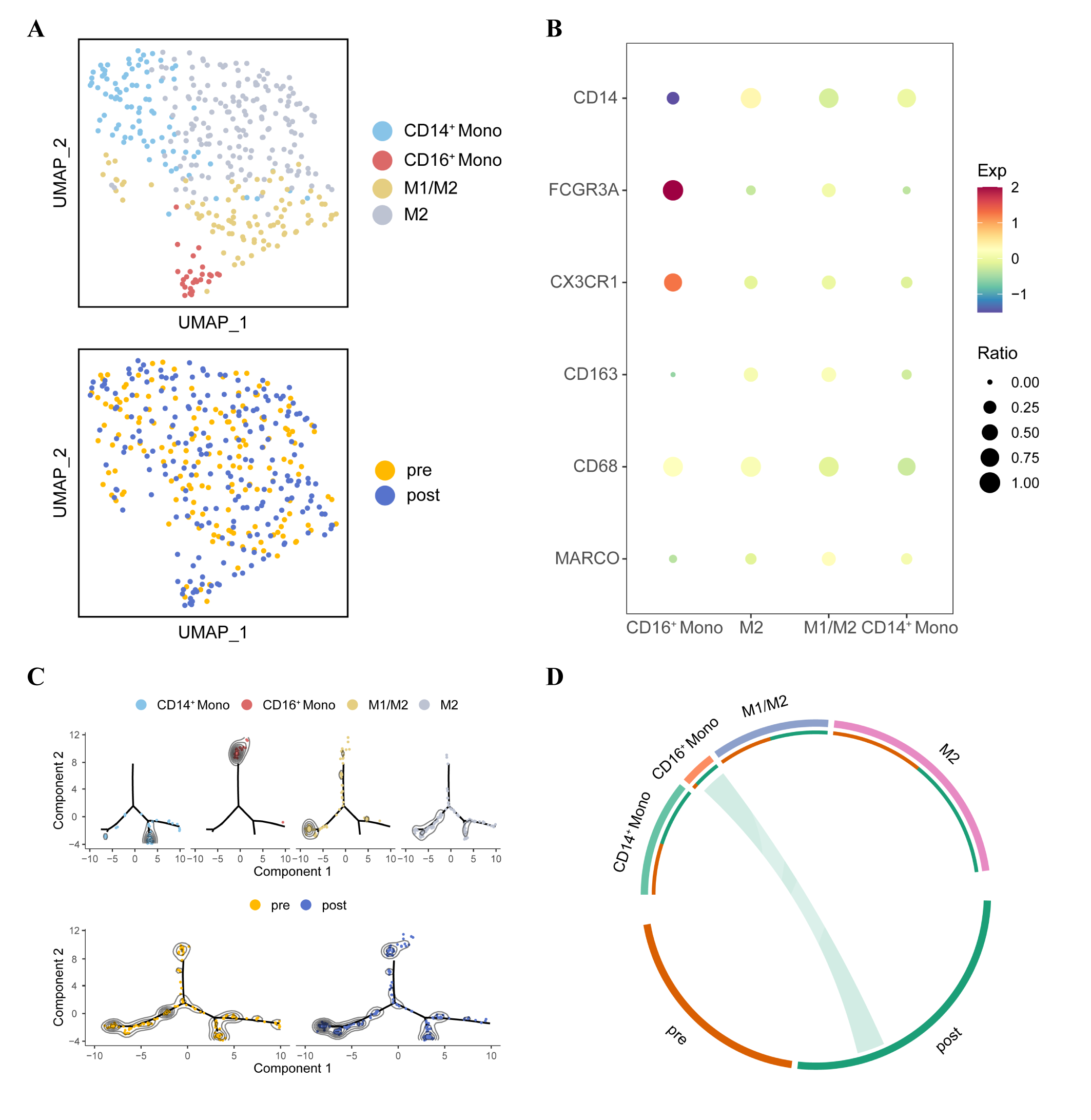

Supplement: Supplementary Figure 5 — Detailed characterization of Myeloid cells and their association with treatment. (A) UMAP of all Myeloid cells, colored by cell type and sample. (B) Bubble plot of selected Myeloid cells function-associated genes in each cell cluster. (C) Pseudotime analysis of Myeloid cells derived from PBMC samples inferred by Monocle2. Each point corresponds to an individual cell colored by cluster (above) or by treatment (down). The density curves represent the distribution of each cluster. (D) The association between subtypes and treatment in Myeloid cells. The area of the ties represents the relative enrichment. [file Image_5.tif]

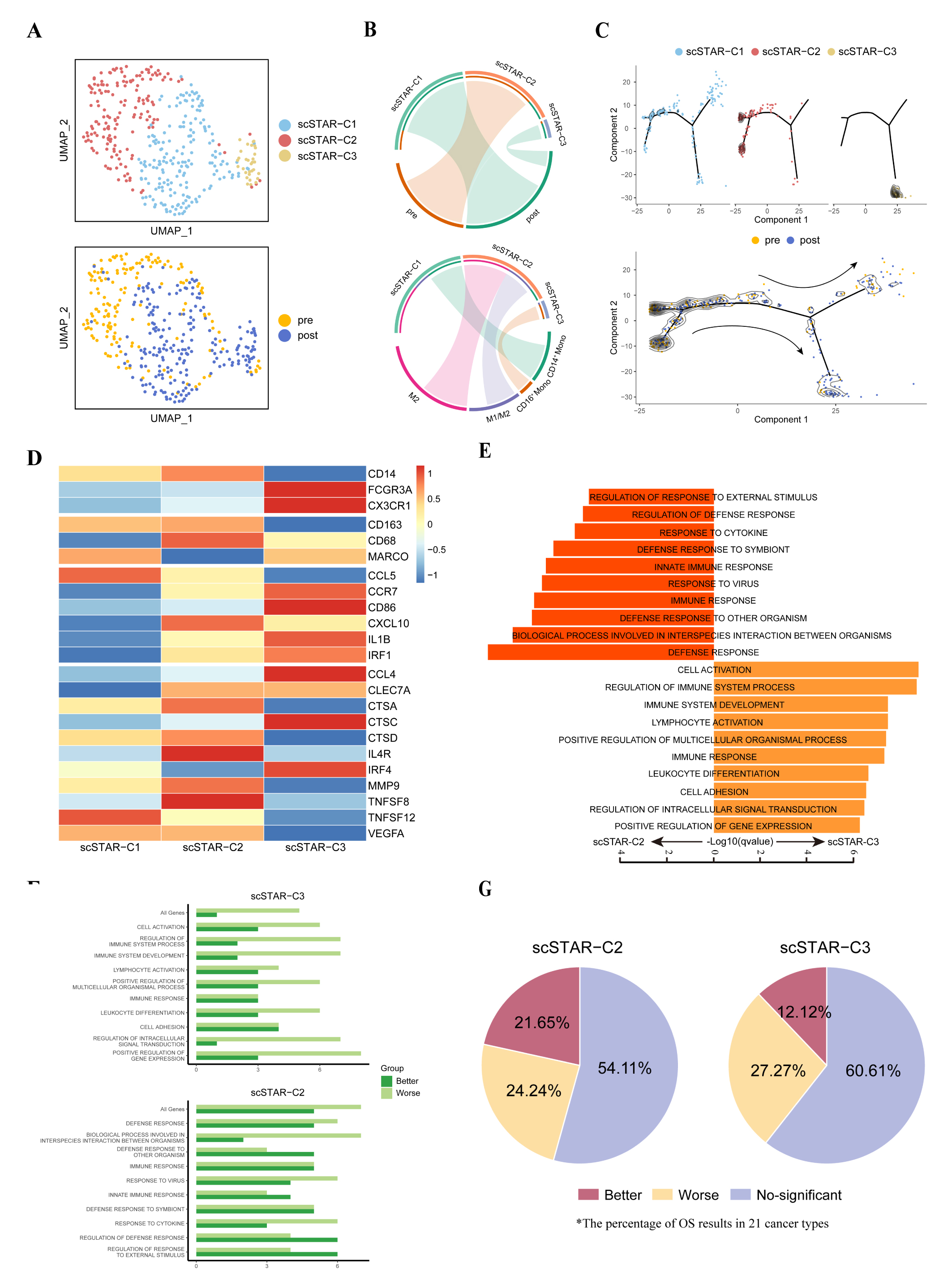

Supplement: Supplementary Figure 6 — The association between molecular functional dynamics of Myeloid cell subsets and prognosis. (A) UMAP of Myeloid cells processed by the scSTAR algorithm, colored by sample and cluster. (B) The association between scSTAR-processed clusters and treatment. The area of the ties represents the relative enrichment. (C) Trajectory analysis for the three scSTAR-processed clusters. Each point corresponds to an individual cell colored by cluster (above) or by sample (down). The density curves represent the distribution of each cluster. (D) Heatmap of scaled normalized expression for scSTAR cell function genes. (E) Pathways enriched in Myeloid cells in scSTAR-C2 and p Myeloid-C3. The bar plot showed the top 10 enriched GO pathways. Benjamini-Hochberg (BH) adjusted p value < 0.05. (F) The number of associations between all changed genes, pathways in which scSTAR-C2 or scSTAR-C3 was involved and better or worse overall survival in the 21 tumor types. (G) The percentage of prognosis results predicted by scSTAR-C2 and scSTAR-C3 in 21 cancer types. [file Image_6.jpg]

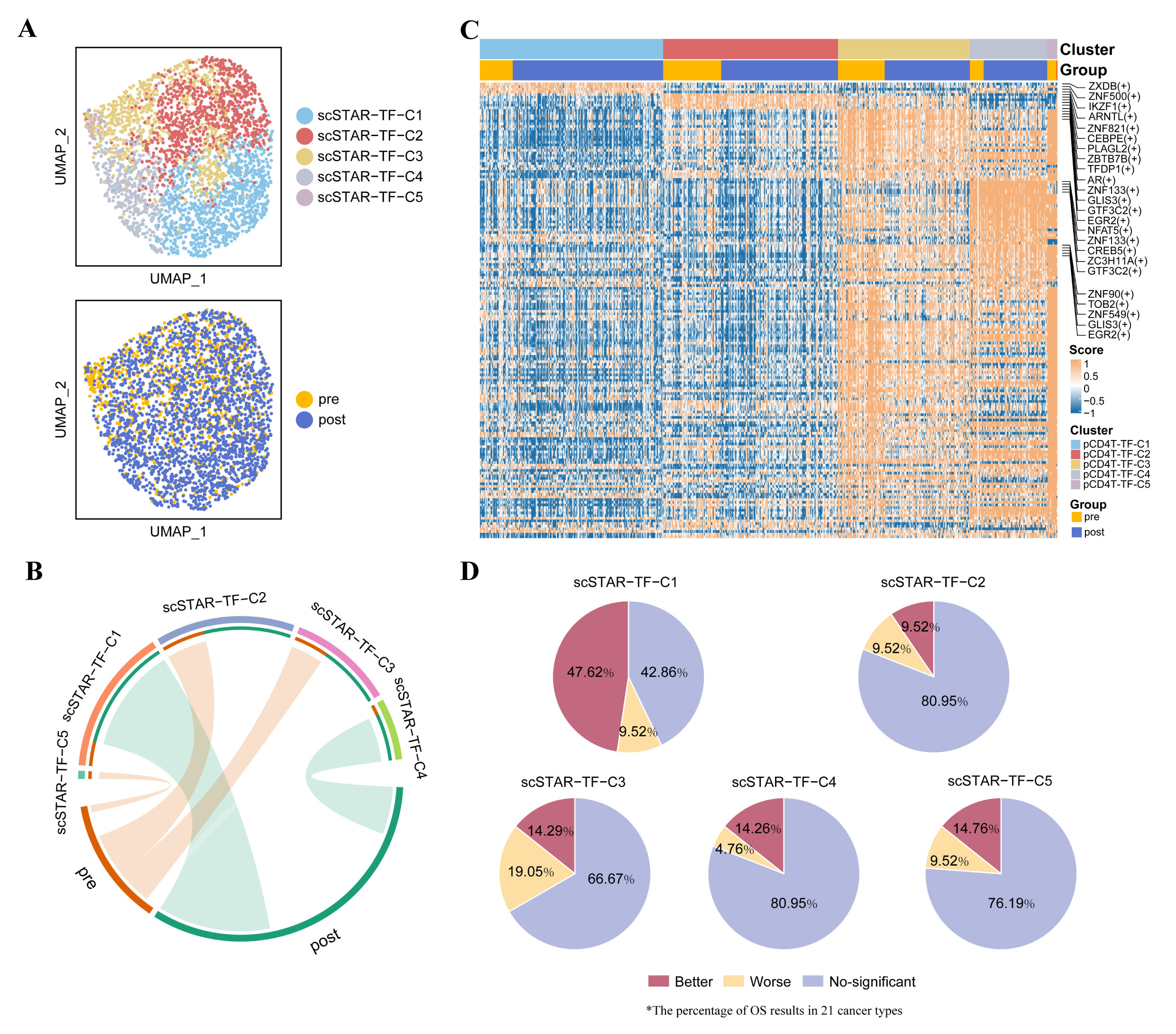

Supplement: Supplementary Figure 7 — scSTAR analysis of gene regulatory networks in CD4+ T cells. (A) UMAP of CD4+ T cells processed by the scSTAR algorithm and SCENIC, colored by sample and cluster. (B) The association between scSTAR-processed clusters and treatment. The area of the ties represents the relative enrichment. (C) Heatmap of scaled normalized regulon activity for CD4+ T cells as determined by two-sided Wilcoxon rank-sum test with Bonferroni FDR correction (q < 0.05). (D) The percentage of prognosis results predicted by scSTAR-processed clusters in 21 cancer types. [file Image_7.tif]

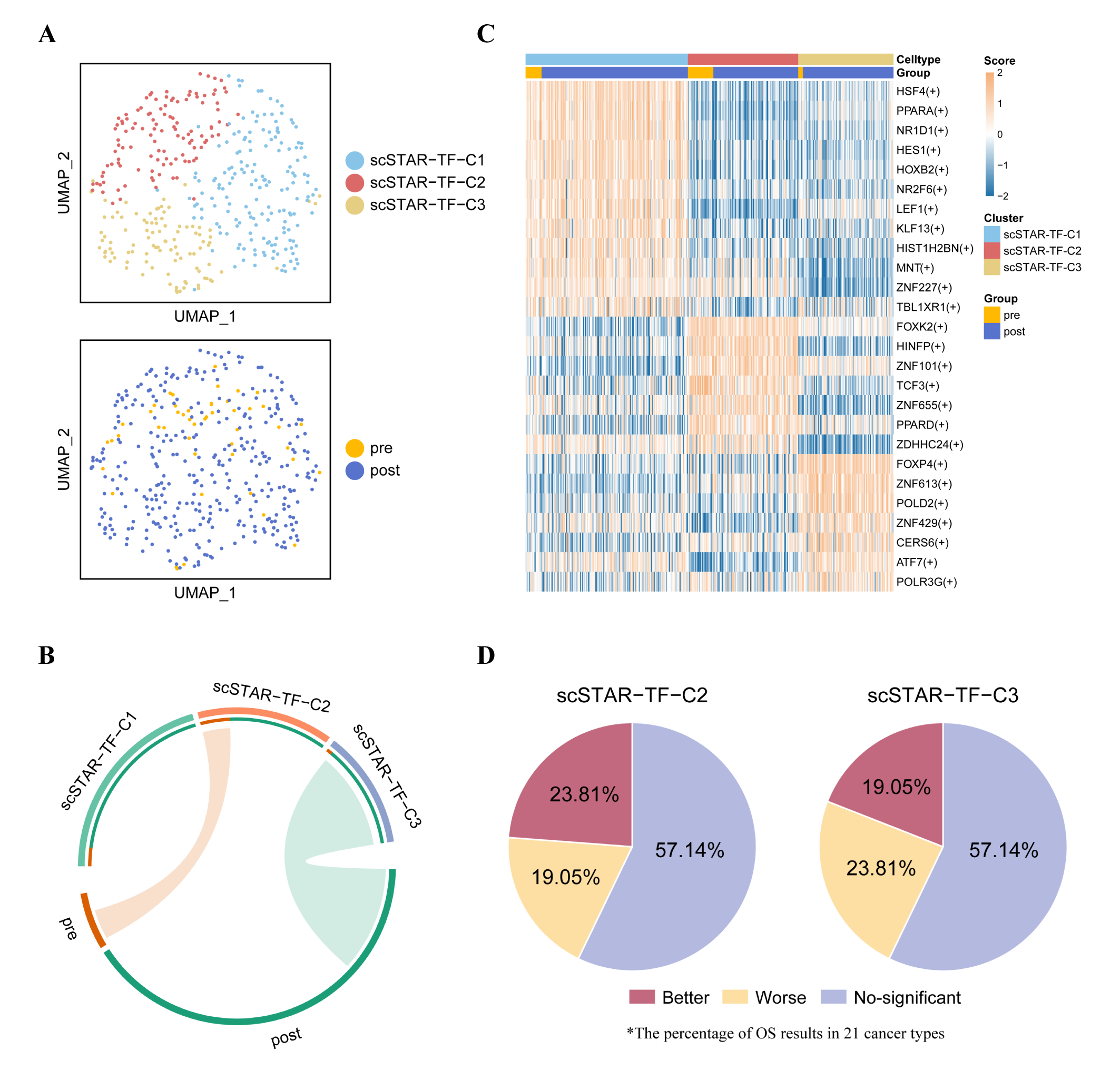

Supplement: Supplementary Figure 8 — scSTAR analysis of gene regulatory networks in B cells. (A) UMAP of B cells processed by the scSTAR algorithm and SCENIC, colored by sample and cluster. (B) The association between scSTAR-processed clusters and treatment. The area of the ties represents the relative enrichment. (C) Heatmap of scaled normalized regulon activity for B cells as determined by two-sided Wilcoxon rank-sum test with Bonferroni FDR correction (q < 0.05). (D) The percentage of prognosis results predicted by scSTAR-processed clusters in 21 cancer types. [file Image_8.tif]
